# Transcriptome-wide N6-methyladenosine modification profiling of long non-coding RNAs during replication of Marek’s disease virus in vitro

**DOI:** 10.1186/s12864-021-07619-w

**Published:** 2021-04-22

**Authors:** Aijun Sun, Xiaojing Zhu, Ying Liu, Rui Wang, Shuaikang Yang, Man Teng, Luping Zheng, Jun Luo, Gaiping Zhang, Guoqing Zhuang

**Affiliations:** 1 College of Veterinary Medicine, Henan Agricultural University, Zhengzhou, 450002 Henan China; 2 Key Laboratory of Animal Immunology, Ministry of Agriculture and Rural Affairs & Henan Provincial Key Laboratory of Animal Immunology, Henan Academy of Agricultural Sciences, Zhengzhou, 450002 China; 3 UK-China Centre of Excellence for Research on Avian Diseases, Henan Academy of Agricultural Sciences, Zhengzhou, 450002 China; 4 College of Animal Science and Technology, Henan University of Science and Technology, Luoyang, 471003 China

**Keywords:** Marek’s disease virus, Long non-coding RNA, m^6^A, MeRIP-Seq, KEGG

## Abstract

**Background:**

The newly discovered reversible N6-methyladenosine (m^6^A) modification plays an important regulatory role in gene expression. Long non-coding RNAs (lncRNAs) participate in Marek’s disease virus (MDV) replication but how m^6^A modifications in lncRNAs are affected during MDV infection is currently unknown. Herein, we profiled the transcriptome-wide m^6^A modification in lncRNAs in MDV-infected chicken embryo fibroblast (CEF) cells.

**Results:**

Methylated RNA immunoprecipitation sequencing results revealed that the lncRNA m^6^A modification is highly conserved with MDV infection increasing the expression of lncRNA m^6^A modified sites compared to uninfected cell controls. Gene Ontology and the Kyoto Encyclopedia of Genes and Genomes pathway analysis revealed that lncRNA m^6^A modifications were highly associated with signaling pathways associated with MDV infection.

**Conclusions:**

In this study, the alterations seen in transcriptome-wide m^6^A occurring in lncRNAs following MDV-infection suggest this process plays important regulatory roles during MDV replication. We report for the first time profiling of the alterations in transcriptome-wide m^6^A modification in lncRNAs of MDV-infected CEF cells.

## Background

Marek’s disease (MD) induced by Marek’s disease virus (MDV) is a lethal lymphotropic disease of chickens that is characterized by severe immunosuppression, neuronal symptoms and the rapid onset of T-cell lymphoma [1]. Based on its genome structure, MDV belongs to the alphaherpesvirus family but nevertheless, the tumorigenic phenotype induced by MDV is more characteristic of gammaherpesviruses [2]. Genome-wide sequencing has revealed that MDV attenuation is related to viral gene mutations [3] and this has been confirmed in vivo through viral gene deletion mutations [4, 5]. Recently however, epigenetic regulatory factors such as DNA methylation and histone modifications have been shown to play important roles in MD [6].

Non-coding RNAs (ncRNAs) constitute a varied group of RNA molecules that do not encode functional proteins. Amongst these are the long non-coding RNAs (lncRNAs), being defined as ncRNAs more than 200 bp long which function as another layer of epigenetic regulation. Moreover, post-transcriptional RNA modifications of lncRNAs may change the expression and activity of mRNAs, ncRNAs and proteins, resulting in epigenetic changes in infected cells. LncRNAs characteristically fulfil regulatory or structural roles in different biological and pathological activities, which are distinct from protein coding genes [7]. For example, the MDV encoded Latency Associated Transcripts (LAT) lncRNA alters the splicing of the viral microRNA (miRNA) cluster to produce indirect effects on host gene expression [8]. Furthermore, the ERL (edited repeat-long) lncRNA edited by Adenosine Deaminase Acting on RNA 1 (ADAR1) is involved in the innate immunity response during virus infection [9]. Expression profiling of long intergenic non-coding RNA (lincRNAs) has also been previously reported in the chicken bursa following MDV infection. Acting through regulation of the *SATB1* gene, the lincRNA linc-satb1 derived from *SATB1* was shown to be crucial in the MDV-induced immune response [10]. Other comprehensive work reporting lncRNA expression profiling indicated that five lncRNAs were strongly related to the expression of MDV and host protein coding genes, and these lncRNAs may play significant roles during MDV-induced tumorigenesis [10]. Among them, linc-GALMD1 inhibited tumor formation through regulating both the expression of MDV and host tumor-related genes [11]. However, whether and how lncRNA expression is regulated during MDV replication is unclear.

Extensive RNA modifications were recently discovered to participate in viral infection through post-transcriptional regulation, decorating both host and viral RNA species. To date, more than 100 distinctive chemical RNA modifications have been identified, including pseudouridine, m^6^A, N1-methyladenosine (m^1^A), and 5-methylcytosine (m^5^C) [12–14]. All of the RNA modifications are mediated by methyltransferase “writer” complex, which is an enzyme complex containing methyltransferase-like 3 (METTL3), METTL4, Wilms’ tumor 1-associating protein (WTPA) and other uncharacterized proteins. Conversely, demethylase complexes include AlkB Homolog 5 (ALKBH5) and FTO which can reverse RNA modifications, acting as an “eraser”. In addition, m^6^A-modified RNAs can be recognized and modulated by the m^6^A-binding protein complex, including YTH N6-Methyladenosine RNA Binding Protein (YTHDF)1, YTHDF2, YTHDF3 and other proteins acting as “readers” [15].

As one of the most abundant and conserved RNA modifications, m^6^A is known to be involved in various viral infections, suggesting an important regulatory role in viral replication and pathogenesis [16]. Here, we performed transcriptome-wide m^6^A modification profiling analyses of lncRNAs, comparing MDV-infected with uninfected chicken embryo fibroblast (CEF) cells. Alterations in the m^6^A signature of lncRNAs suggests that m^6^A modifications may play important regulatory roles during MDV replication.

## Results

### Transcriptome-wide m^6^A modifications in lncRNAs after Md5 (a very virulent MDV strain) infection

**Fig. 1:**
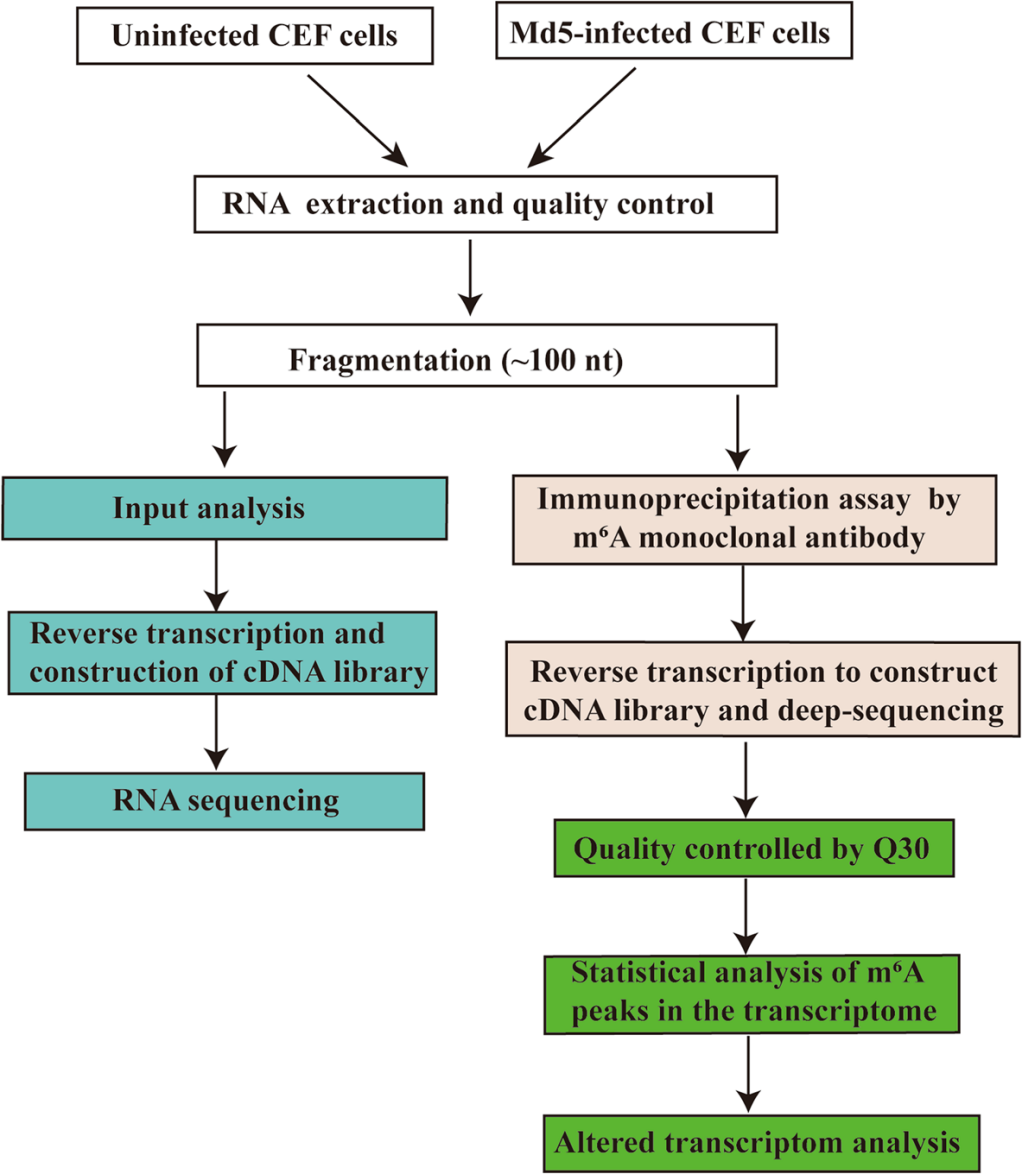
Flowchart illustrating the construction of cDNA libraries used for RNA sequencing

**Fig. 2:**
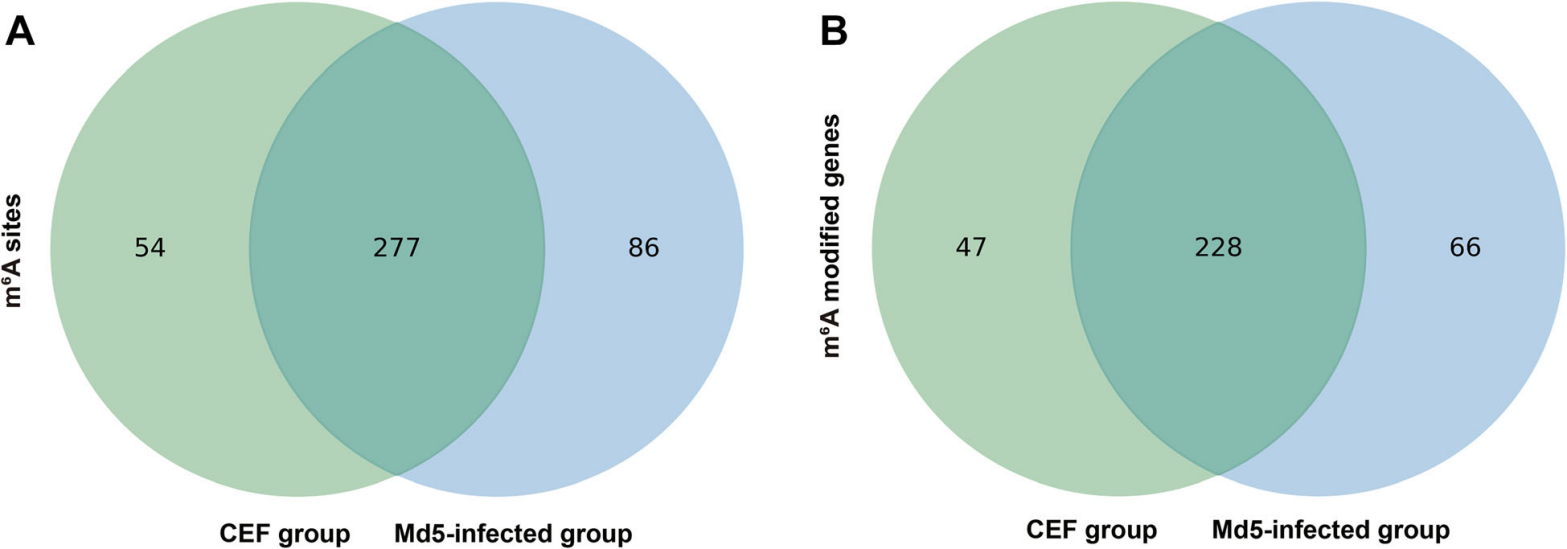
Transcriptome-wide m^6^A modifications in lncRNAs following Md5 infection. **a** Venn diagram of m^6^A modification sites identified in lncRNAs from mock control and Md5-infected groups; **b** Venn diagram of m^6^A modified lncRNAs from mock control and Md5-infected groups

RNA-sequencing and transcriptome analyses were performed on mock control and Md5-infected CEF cells following successful construction of cDNA libraries (Fig. 1). To gain further information of transcriptome-wide m^6^A modifications in the lncRNAs, we then performed Methylated RNA immunoprecipitation sequencing (MeRIP-seq). Altering the m^6^A sites with fold changes (FCs) > 2 was considered to be unique to specific sites. Using this approach, we identified 363 and 331 m^6^A peaks in the Md5 and control groups, respectively (Fig. 2a). Furthermore, a total of 294 and 275 annotated genes were mapped to the Md5-infected and control groups, respectively (Fig. 2b). Among them, 277 m^6^A peaks and 228 m^6^A modified genes were detected in both the Md5-infected and control groups. Overall, these results indicated that the incidence of the m^6^A modification in lncRNAs was higher in the Md5 infected group compared to the control group.

### m^6^A modification clustering analysis

**Fig. 3:**
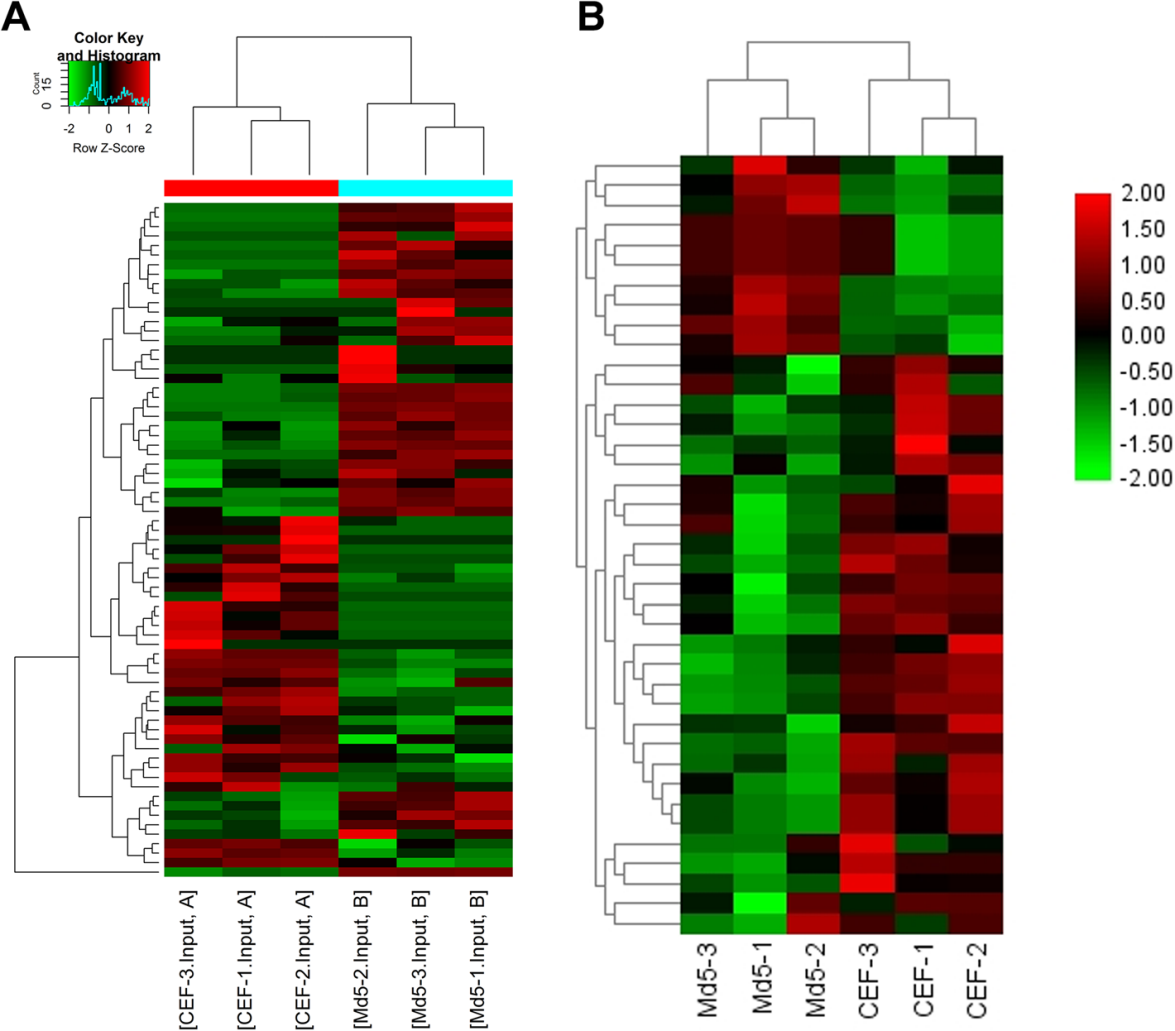
m^6^A modification clustering analysis. Cluster analysis of the transcriptome (**a**) and m^6^A modified lncRNA genes (**b**) in mock control and Md5-infected groups. The color intensity represents the size of the log-fold enrichment (FE) value; the closer the color is to red, the larger the logFE value

Results from the methylation heat map and cluster analysis showed that the different clustering could clearly distinguish the m^6^A modification at the transcriptome level in the Md5-infected group from the control group (Fig. 3a). These findings indicate that the degree of methylation in the Md5-infected group was significantly higher than for the control group (Fig. 3b). In total, 70 m^6^A modification peaks were identified as being up-regulated (Table 1) with 53 methylation peaks being down-regulated amongst lncRNA genes (Table 2).

**Table 1:** Ten top up-methylated m^6^A peaks

Chromsome	TxStart	TxEnd	Gene name	Fold change
AADN04013810.1	1301	1455	ENSGALG00000046022	450
AADN04002949.1	13515	13760	ENSGALG00000035221	344.6
AADN04002949.1	11941	12259	ENSGALG00000035221	264.4
1	32604032	32604254	LOC107052719	128.74691
AADN04002878.1	4081	4300	ENSGALG00000041302	73.769912
KQ759420.1	20181	20501	ENSGALG00000037624	71.7
AADN04004826.1	2661	2880	ENSGALG00000031041	66.348837
AADN04013810.1	2275	2800	ENSGALG00000046022	52.521739
AADN04016904.1	1271	1660	ENSGALG00000038053	36.209302
13	16955381	16955734	LOC100857928	15.307692

Notes: Chromsome/ TxStart/ TxEnd: the coordinates of the differentially methylated RNA sites in bed format, please ref http://genome.ucsc.edu/FAQ/FAQformat.html#format1.

Gene name: the gene ID assigned by stringtie.

Fold change: fold change between two groups.

**Table 2:** Ten top down-methylated m^6^A peaks

Chromsome	TxStart	TxEnd	Gene name	Fold change
AADN04015281.1	281	580	ENSGALG00000030158	87.5
1	85973346	85973760	ENSGALG00000037227	62.4
AADN04009117.1	7101	7460	ENSGALG00000032284	15.2059801
AADN04014355.1	1841	2140	ENSGALG00000033167	11.4929742
AADN04009117.1	5824	6280	ENSGALG00000032284	8.06483791
AADN04003477.1	14181	14181	ENSGALG00000031733	7.98591549
AADN04013890.1	4221	4800	ENSGALG00000037255	7.1248074
Z	144568	144700	LOC101751186	6.08733624
1	17382500	17382545	ENSGALG00000039093	6.08306709
AADN04006665.1	10281	10473	ENSGALG00000039244	4.73853211

Notes: Chromsome/ TxStart/ TxEnd: the coordinates of the differentially methylated RNA sites in bed format, please ref. http://genome.ucsc.edu/FAQ/FAQformat.html#format1

Gene name: the gene ID assigned by stringtie

Fold change: fold change between two groups

### Chromosome visualization of m^6^A modified lncRNAs

**Fig. 4:**
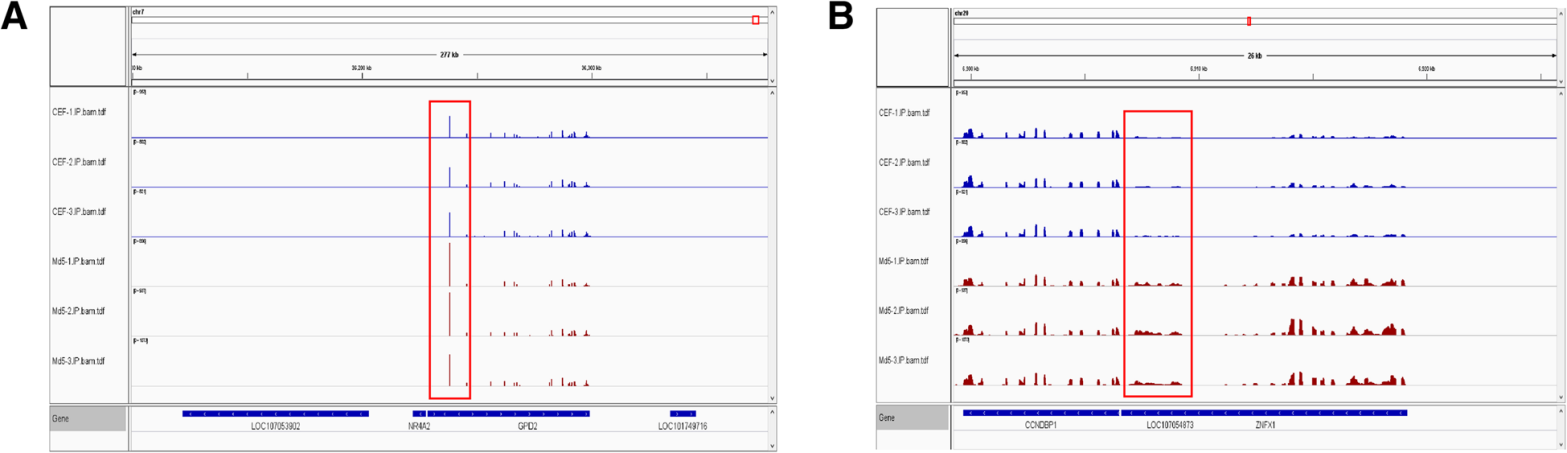
Differentially methylated N6-methyladenosine peaks in lncRNAs. Both **a** and **b** showed that representative upmethylated genes in Md5-infected group relative to mock control group. Highlighted columns show the general locations of differentially methylated peaks

Studying the genomic distribution of m^6^A methylation sites revealed that lncRNA genes undergoing the m^6^A modification were scattered on all chromosomes. However, the methylation levels and distribution of m^6^A of lncRNA genes on each chromosome were different between infected and control groups, a finding which may functionally associate m^6^A with MDV infection (Fig. 4a and b).

### Abundance of m^6^A peaks and conserved m^6^A modified motifs in lncRNAs

**Fig. 5:**
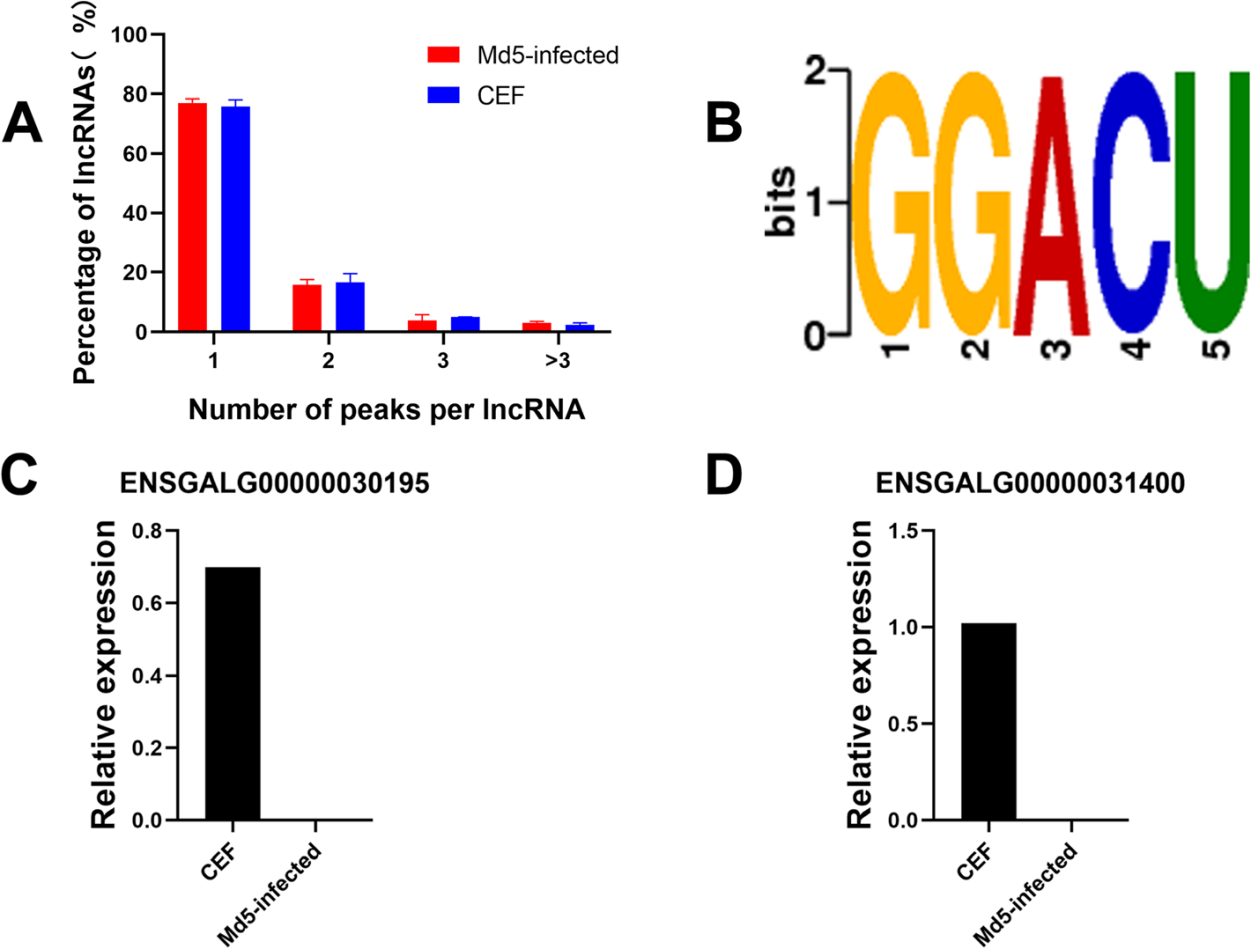
Abundance of m^6^A peaks and the conserved m^6^A modified motif in lncRNAs. **a** Number of lncRNA harboring different numbers of m^6^A peaks in the two groups, with the majority harboring only one m^6^A peak; **b** The sequence motif of m^6^A sites in Md5-infected and mock control groups; MeRIP-qPCR analysis of two candidate lncRNAs **c** ENSGALG00000031400 and **d** ENSGALG00000030195. * and ** respectively represent the significant difference in gene expression between two groups (* for *P*-value < 0.05 and ** for *P*-value < 0.01)

Regarding the abundance of the m^6^A peaks in lncRNAs, we found that 77.13% of the lncRNAs in the Md5-infected group contained m^6^A peaks, which appeared marginally more than the unimodal value calculated at 75.86% in the control group. The respective percentages comparing different numbers of peaks were also determined with two peaks, three peaks, and more than three peaks being 15.81 vs 16.66, 3.92% vs 5.10 and 3.14% vs 2.38%, respectively, for the Md5 infected versus control group (Fig. 5a).

To analyze the conserved motif of m^6^A modified lncRNAs, we selected the sequences of the first 1000 peaks with the highest enrichment factor in each group (50 bp on both sides of the peak), and scanned the sequences of these peaks using DREME software [17] to determine whether the identified m^6^A peak contained the RRACH conservative motif sequence (where R represents purine, A represents m^6^A and H represents non-guanine bases). The sequence of the top ten peaks with the highest enrichment ratio of lncRNA (50 bp on each side of the vertex) was compared with the motif sequence found, and it was found that GGACU sequence was one of the conserved motif sequences of lncRNA (Fig. 5b). GGACU is one of the motif obtained based on E-value. For the peak with GGACU sequence in control group is 202/1000 (202 peaks out of 1000 peaks used for analysis contain this sequence). In Md5-infected group it was 165/1000.

To further confirm the existence and distinctive expression of m^6^A modified lncRNAs. The relative expression of two lncRNAs were confirmed by m^6^A methylated RNA immunoprecipitation-qPCR (MeRIP-qPCR) (Fig. 5c and d). The results indicated that the results of MeRIP-qPCR are consistent with RNA-Seq.

### GO enrichment analysis

To explore the potential function of m^6^A in CEF cells and infected cells, we carried out GO enrichment analysis of differentially m^6^A-methylated genes of lncRNAs. The GO Project has developed a structured, controlled vocabulary for annotating genes, gene products and sequences divided into three parts: molecular function (MF), biological process (BP) and cellular component (CC). GO function analysis performed against the differentially methylated lncRNAs showed no significant enrichment but when analysis was performed on the input sequencing data, only the up-regulated methylated sites were found.

**Fig. 6:**
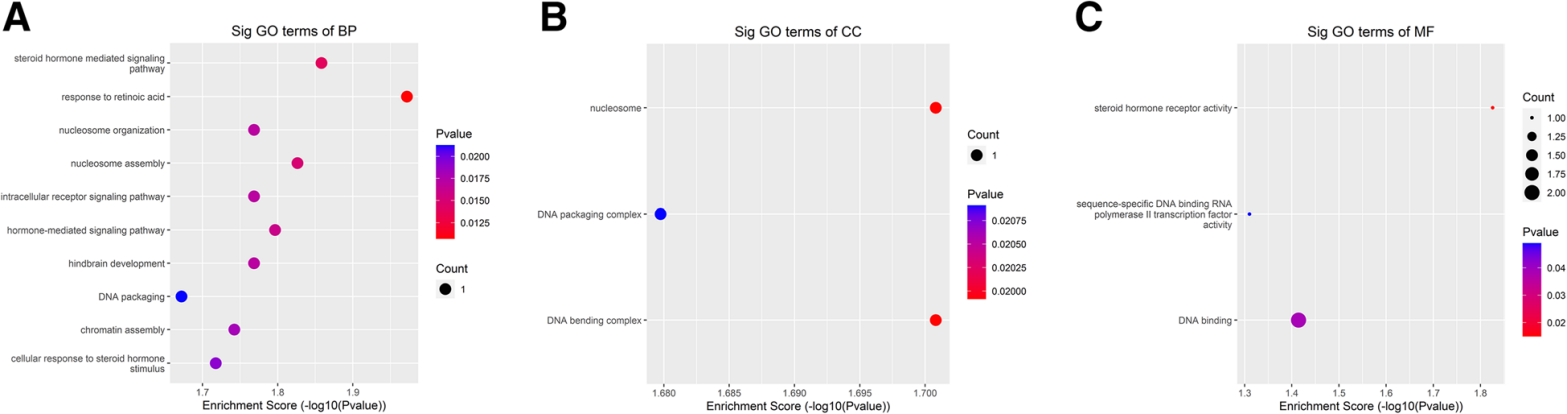
GO analysis of coding genes harboring differentially methylated m^6^A sites. The top ten GO terms for **a** biological processes; **b** molecular functions; and **c** cellular components significantly enriched for the up-methylated transcriptome in Md5-infected versus mock control groups

The BP data showed enrichment in steroid hormone receptor activity, sequence-specific DNA binding RNA polymerase II transcription factor activity and DNA binding (Fig. 6a). CC data showed mainly enrichment for nucleosome, DNA packaging complex and DNA bending complex (Fig. 6b). The MF outputs showed the genes with increased methylation were notably enriched in the steroid hormone mediated signaling pathway, response to retinoic acid, nucleosome organization, nucleosome assembly, hindbrain development, DNA packaging, chromatin assembly and cellular response to steroid hormone stimulus (Fig. 6c).

### KEGG pathway analysis

**Fig. 7:**
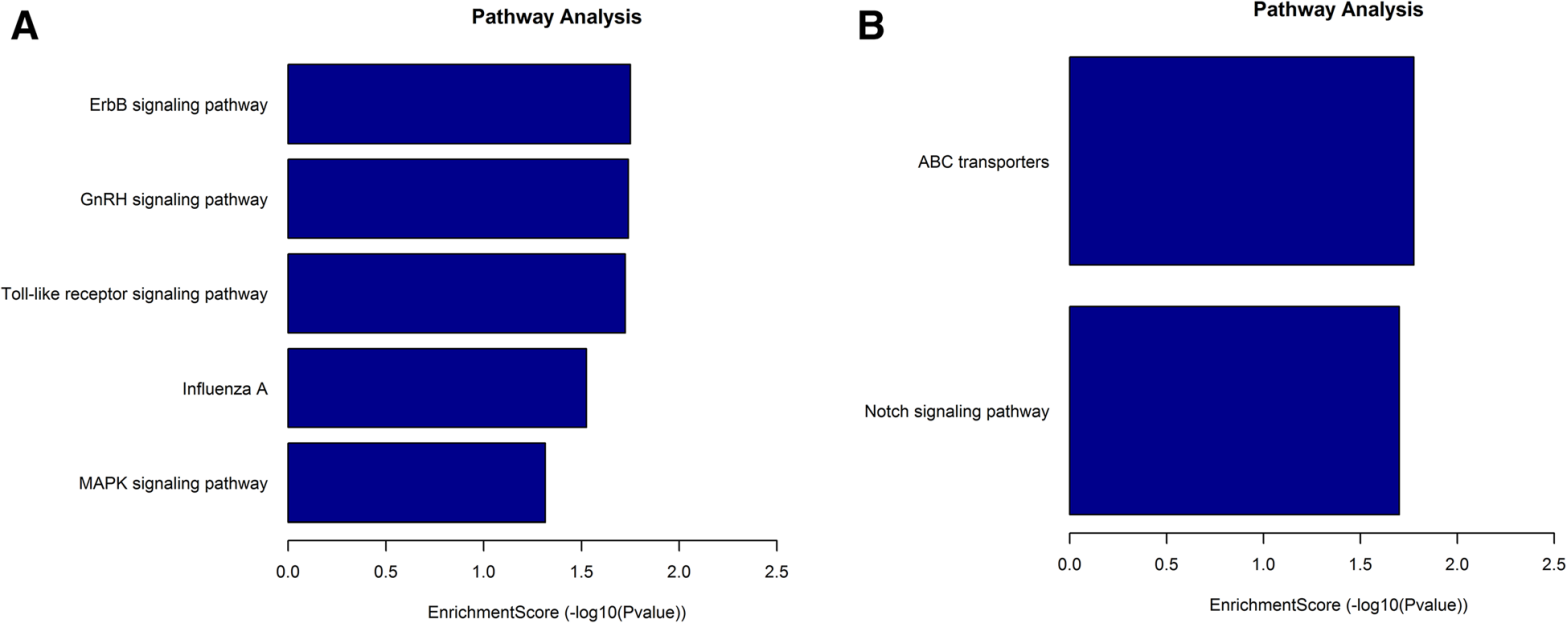
KEGG analysis and gene set enrichment analysis (GSEA) of differentially methylated genes in Md5-infected and control groups; **a** Pathway analysis of up-methylated; **b** down-methylated genes

KEGG analyses map molecular data sets from genomics, transcriptome, proteomics and metabolomics to explore associated biological functions. KEGG pathway analyses indicated significant gene enrichments associated with five up-regulated pathways, including ErbB signaling, GnRH signaling and Toll-like receptor signaling pathways along with Influenza A and MAPK signaling (Fig. 7a). Two significantly down-regulated pathways involved ABC transporters and Notch signaling (Fig. 7b).

## Discussion

### The transcriptome-wide m^6^A modification is important in virus infection

MD is a highly contagious tumor-causing disease which threatens all poultry-raising countries across the globe [18]. The pathogenesis of MD is complex with apparent genetic changes, heritable gene expression changes and chromatin tissue being shown to promote tumor initiation and progression. Additionally, it is now emerging that epigenetic changes, particularly those associated with reversible chemical modifications of RNA, fulfil important roles in the life cycle of viruses and therefore also in viral pathologies. For example, HIV infection increases the levels of m^6^A modification in both viral and host transcripts, and moreover, m^6^A modified-HIV transcripts display enhanced binding ability to viral proteins. Instructively, knockdown of the ALKBH5 demethylase or alternatively the METTL3/14 methylase to alter the level of HIV m^6^A modifications either promotes or inhibits viral replication, respectively [19]. Furthermore, twelve m^6^A modified sites have been found in ZIKV genomic RNA but in contrast to HIV, demethylase knockout inhibits ZIKV replication, while methylase knockout increases ZIKV replication rates. However, the impact of the m^6^A modification in MVD is yet to be determined [20].

### MDV infection increased lncRNAs m^6^A modification

In the present study, we investigated how the m^6^A modification in lncRNAs was affected by MDV infection. The results obtained in CEF cells showed that the abundance and distribution of m^6^A in Md5-infected and control groups were different albeit not significantly. Interestingly, we found that some of the lesser expressed genes in the control group were not only highly expressed in the infected group, but also displayed increased levels of m^6^A modification. Interestingly, there were significantly higher expressions of METTL14 and ALBHK5 in MDV infected CEF cells comparing to mock-infected control (Data not shown). This suggests MDV might control lncRNAs m^6^A modification through regulating activities of methyltransferase and demethylase, and even reader proteins. It is of great importance to determine the detailed mechanism of how MDV affect and regulate the lncRNAs m^6^A modification in the future. Alternatively, the role of m^6^A modified lncRNAs on MDV replication also need to be further investigated.

### MDV infection altered lncRNAs m^6^A modification associated with genes function

GO analysis of the m^6^A modified genes showed that most are up-regulated methylated sites. For BP, CC and MF, up-regulated methylated genes were notably enriched in steroid hormone mediated signaling pathway, nucleosome organization, nucleosome assembly, DNA packaging, DNA binding complex, chromatin assembly and cellular response to steroid hormone stimulus. Most of these biological activities are related to virus replication, suggesting lncRNA may change structural and regulatory roles after m^6^A modification.

### MDV infection altered lncRNAs m^6^A modification associated with signaling pathways

LncRNA expression can be variously regulated by histone modification, DNA methylation or through changes in the expression of the responsible transcription factors. In this study, many differentially expressed m^6^A modification sites were found, among which the unique m^6^A modification related genes were only found in Md5-infected group. These results suggest that some of the m^6^A modification sites are changed by Md5 virus infection. Furthermore, KEGG pathway analyses implicate roles for m^6^A-modified lncRNAs in biological pathways known to be associated with viral infection, namely ErbB signaling, GnRH signaling, Toll-like receptor signaling, Influenza A and the MAPK signaling pathway. Notably the ErbB gene encoding tyrosine kinases of the epidermal growth factor (EGF) receptor family can promote herpesvirus replication [21] while the Toll-like receptor signaling pathway is also upregulated by MDV infection in vitro [22]. The mitogen-activated protein kinase (MAPK) upstream of intracellular signaling pathways also participates in HSV-1 cell-to-cell spreading. Indeed, MDV infection alters MAPK signaling in vitro and in vivo, suggesting a key role in herpesvirus replication and even pathogenesis [23, 24]. Furthermore, influenza A virus (IAV) infection activates multiple signaling pathways to overcome the innate immunity barrier where IAV is recognized by the pathogen recognition receptor RIG-I to control type I IFN production [25]. Notably, it has been demonstrated that AIV expresses m^6^A modified transcripts and that inhibition of m^6^A could decrease gene expression and inhibit AIV replication [26]. Moreover, mutations in AIV transcripts to alleviate m^6^A modifications reduced viral pathogenicity thereby confirming this important regulatory role. Thus overall, there is evidence that up-regulation of m^6^A modified transcripts might be a common feature for both DNA and RNA viruses that helps facilitate viral replication through regulating host RNA regulatory pathways [27].

## Conclusions

In this study, we employed MeRIP-seq to evaluate differential lncRNA m^6^A modifications following Md5 infection. Comparing MDV infected and control cells we identified the abundance of m^6^A modifications and the genome wide utilization of the conserved motif. Tellingly, we observed increased lncRNA m^6^A modifications following Md5 infection, clearly suggesting a relationship between lncRNA m^6^A modifications and viral infection. In support, GO and KEGG analyses showed genes with up-regulation of methylation were associated with host cell signaling pathways known to contribute to viral infection. However, further investigations are required to dissect the molecular mechanisms linking m^6^A-modified lncRNAs with MDV pathogenesis and tumorigenesis.

## Methods

### Cells and virus

CEF cells were isolated and prepared from 9-day-old specific-pathogen-free (SPF) embryonated white leghorn chicken (Boehringer Ingelheim, Beijing, China) as previously described [28]. CEF cells were maintained in Dulbecco’s modified essential medium (DMEM) (Solarbio, Beijing, China) containing 5% fetal bovine serum (FBS) (Gibco, CA, USA).

A very virulent MDV strain, Md5 (Genbank accession no: NC_002229.3) was used in the present study. For virus infection assay, secondary CEF cells were seeded to 80–90% confluence in T75 culture dishes and separated into mock-infected and infected groups with three repeats in each group. The infected group was inoculated with 10^6^ plaque formation units (PFU) of the Md5 strain (passage two) and cells harvested 7 days post-inoculation when the cytopathic effects (CPE) became clearly visible in about 80% of infected cells.

### RNA extraction

Total RNA was extracted using Trizol reagent (Invitrogen Corporation, Carlsbad, CA) according to the manufacturer’s instruction, with DNase treatment. RNA concentrations were quantified using a Nanodrop ND-1000 spectrophotometer (Thermo Fisher Scientific, Waltham, MA, USA).

### cDNA library construction

RNA samples were fragmented into 100 bp using fragmentation buffer and then incubated with anti-m^6^A polyclonal antibody (Synaptic Systems, 202,003, Germany) in immunoprecipitation (IP) buffer for 2 h at 4 °C. The mixture was then immunoprecipitated by incubation with protein-A beads (Thermo Fisher Scientific, Waltham, MA, USA) at 4 °C for an additional 2 h. Then, bound RNA was eluted from the beads with N6-monophosphate (BERRY & ASSOCIATES, PR3732) in IP buffer and then extracted with Trizol reagent. Purified RNA was used for RNA-seq library generation with NEBNext® Ultra™ II Directional RNA Library Prep Kit (New England Biolabs, USA) following the manufacturer’s instructions. Both the input sample without immunoprecipitation and the m^6^A IP samples were subjected to 150 bp paired-end sequencing on an Illumina HiSeq 4000 sequencer [14].

### Sequencing and data analysis

Paired-end reads were harvested for image and base recognition with Q30 used as the quality control standard, with the sequencing quality of Q30 being usually over 80%. After 3′ adaptor-trimming and low-quality reads removing by cutadapt software (v1.9.3), the reads were aligned to the chicken reference genome (Gal5; GCA_000002315.3) with Hisat2 software (v2.0.4). The expressed lncRNAs were identified using Input reads and the methylated sites on lncRNAs identified using the MeTPeak package in R software. Differentially methylated sites were identified by MeTDiff package in R. The Gene Ontology (GO) (http://www.geneontology.org) and pathway enrichment analysis were performed for the differentially methylated genes. The read alignments on genome were visualized using the interactive analysis tool Integrative Genomics Viewer (IGV).

To define the possible roles of the differentially methylated genes, the GO functions were analyzed using the corresponding lncRNA genes as inputs. GO terms providing *P*-values ≤0.05 were considered to be statistically significant. In concert, Kyoto Encyclopedia of Genes and Genomes (KEGG) [29] analyses of the genes associated with differentially methylated lncRNAs were used as inputs to derive significantly altered pathways. *P*-values < 0.05 were taken as the threshold for significant enrichment.

### m^6^A methylated RNA immunoprecipitation-qPCR (MeRIP-qPCR)

We selected two differentially methylated RNA sites (ENSGALG00000031400 and ENSGALG00000030195) to design specific primers for MeRIP-qPCR using NCBI Primer-Blast [30]. The forward primer (5′-TCATGGCCTGATTCTTTGAGC-3′) and reverse primer (5′-TGCTGTGGATTGGCTTGGAA-3′) designed to amplify 100 bp of ENSGALG00000031400, and the forward primer (5′-CAGCTGCCTGAACAAGGAGA-3′) and reverse primer (5′-ACATACTGCTAAAGCTCAGGAA-3′) designed to amplify 101 bp of ENSGALG00000030195 were synthesized by Sangon Biotech Co. (Shanghai, China). Then reverse transcribed IP RNA and input RNA by PrimeScriptTM RT Reagent Kit and gDNA Eraser Kit (TAKARA, Shiga, Japan) to get cDNA, and qPCR was performed on QuantStudio™ 5 System.

## Data Availability

All data generated or analyzed during this study are included in this submitted manuscript. The datasets generated and/or analyzed during the current study are available in the NCBI repository (https://www.ncbi.nlm.nih.gov/geo/). The data is accessible via NCBI GEO submission ID: GSE166240. To review GEO accession GSE166240: Go to https://www.ncbi.nlm.nih.gov/geo/query/acc.cgi?acc=GSE166240. Enter token klufyeaednulxgb into the box.

## Abbreviations

MDV: Marek’s disease virus
MD: Marek’s disease
m^6^A: N6-methyladenosine
CEF: Chicken embryo fibroblast
MeRIP-Seq: Methylated RNA immunoprecipitation sequencing
GO: Gene ontology
KEGG: Kyoto encyclopedia of genes and genomes
ADAR1: Adenosine Deaminase Acting on RNA 1
LAT: Latency Associated Transcripts
lincRNA: long intergenic non-coding RNA
UL: Unique long region
US: Unique short region
MDV-1: MDV serotype 1
MDV-2: MDV serotype 2
MDV-3: MDV serotype 3
ncRNAs: non-coding RNAs
miRNAs: microRNAs
lncRNAs: long non-coding RNAs
SPF: Specific pathogen-free
METTL3: Methyltransferase like protein 3
METTL14: Methyltransferase like protein 14
WTAP: Wilms’ tumor 1-associating protein
FTO: Fat mass and obesity-associated protein
ALKBH5: AlkB homolog 5 RNA demethylase
YTH: YT521-B homology
mRNA: messenger RNA
m1A: N1-adenylate methylation
m5C: Cytosine hydroxylation
SPF: Specific pathogen free
EGF: Epidermal growth factor
MAPK: Mitogen-activated protein kinase
IAV: influenza A virus
DMEM: Dulbecco’s modified essential medium
FBS: Fetal bovine serum
PFU: Plaque formation units
CPE: Cytopathic effects
IP: Immunoprecipitation
IGV: Integrative Genomics Viewer
BP: Biological processes
MF: Molecular functions
FC: Fold change
CC: Cellular components
FE: Fold enrichment
